# Ten grams and 13,000 km on the wing – route choice in willow warblers *Phylloscopus trochilus yakutensis* migrating from Far East Russia to East Africa

**DOI:** 10.1186/s40462-018-0138-0

**Published:** 2018-10-15

**Authors:** Kristaps Sokolovskis, Giuseppe Bianco, Mikkel Willemoes, Diana Solovyeva, Staffan Bensch, Susanne Åkesson

**Affiliations:** 1 Department of Biology, Center for Animal Movement Research, Lund University, Ecology Building, 223 62 Lund, SE Sweden; 2 Department of Biology, Molecular Ecology and Evolution Laboratory, Lund University, Ecology Building, 223 62 Lund, SE Sweden; 3 Institute of Biological Problems in the North, Magadan, Russia; 4 Department of Biology, Evolutionary Ecology Unit, Lund University, Ecology Building, 223 62 Lund, SE Sweden

**Keywords:** Bird migration, Compass orientation, Range expansion, Route simulations

## Abstract

**Background:**

High-latitude bird migration has evolved after the last glaciation, in less than 10,000–15,000 years. Migrating songbirds rely on an endogenous migratory program, encoding timing, fueling, and routes, but it is still unknown which compass mechanism they use on migration. We used geolocators to track the migration of willow warblers (*Phylloscopus trochilus yakutensis*) from their eastern part of the range in Russia to wintering areas in sub-Saharan Africa. Our aim was to investigate if the autumn migration route can be explained by a simple compass mechanism, based on celestial or geomagnetic information, or whether migration is undertaken as a sequence of differential migratory paths possibly involving a map sense. We compared the recorded migratory routes for our tracked birds with simulated routes obtained from different compass mechanisms.

**Results:**

The three tracked males were very similar in the routes they took to their final wintering sites in southern Tanzania or northern Mozambique, in their use of stopover sites and in the overall timing of migration. None of the tested compass mechanisms could explain the birds’ routes to the first stopover area in southwest Asia or to the destination in Southeast Africa without modifications. Our compass mechanism simulations suggest that the simplest scenarios congruent with the observed routes are based on either an inclination or a sun compass, assuming two sequential steps.

**Conclusions:**

The birds may follow a magnetoclinic route coinciding closely with the tracks by first moving west, i.e. closer to the goal, and thereafter follow a constant apparent angle of inclination to the stopover site. An alternative would be to use the sun compass, but with time-adjustments along the initial part of the migration to the first stopover, and thereafter depart along a new course to the winter destination. A combination of the two mechanisms cannot be ruled out, but needs to be confirmed in future studies.

**Electronic supplementary material:**

The online version of this article (10.1186/s40462-018-0138-0) contains supplementary material, which is available to authorized users.

## Background

Evolution of spectacular long-distance migration has occurred multiple times across taxa [12]. The distances and form of migratory routes followed by animals are shaped by a number of ecological factors, such as physiological capacity, transportation cost, competition, food availability, continental outline, topography, barriers, winds and navigational mechanisms [12]. Current migratory routes to the most northerly breeding latitudes, have evolved over short time periods dating back no longer than the Pleistocene, following the last glaciation 10,000 to 15,000 years BP (e.g. [20, 55]). Some regions, such as northeastern Siberia, were not fully glaciated, and may therefore have been accessible for terrestrial birds throughout the last glaciation period [53]. However, it is unlikely that Beringia was used by migratory species having wintering grounds in Africa, as this would have required them to migrate across vast glaciated territories [18]. The evolution of new migratory routes is expected to be formed by gradual range expansion and colonization events of distant northern landmasses as the ice retreated (e.g. [20, 26, 36, 42, 44, 60]), but it is still open if any known compass mechanisms may be part of this process [32]. Performance of long migrations, and to timely arrive to explore distant resources available during limited time, however, are challenging for migratory birds involving sophisticated diurnal and circannual time-keeping [7]. Still many birds are capable of extremely long migrations, of which the willow warbler performs one of the longest among songbirds, weighing less than 10 g and migrating > 10,000 km one-way in one season [12].

Most songbirds migrate alone, and their migratory program encodes timing, prolonged periods of continuous flight, fueling, but also adaptations for long flights and barrier crossings, selection of flight altitudes and navigation capabilities [5]. All these phenotypic attributes provide individual songbirds with genetically encoded population-specific migrational adaptations and route preferences [21, 37–39, 48, 63]. The migration phenotype inherited from successful parents will enable individuals to successfully migrate alone from where they were born to the population-specific wintering sites and back [17]. Songbirds have access to several biological compasses based on information from the sun, the stars and the Earth’s magnetic field for compass orientation (e.g. [1, 6, 30, 76]. Specifically, they have inherited a migratory direction and information on for how long time they should be active to reach the destination during the first migration [17, 33, 41]. Encoded may also be shifts in migratory courses [16, 34, 40], and where to fuel before barrier crossings [31] along the route, for which predicted geomagnetic information needs to be experienced to trigger a behavioural or physiological change. To manage migratory flights, also coordination of compass cues during development [72], compass calibration ([57], cf. [10]), and course-correction mechanisms (e.g. longitude detection; [9]) will be needed. From the first to later migrations, additional map cues may further be incorporated in the migration program expressed in adult birds [62].

The complexity of migratory routes may be affected by historic colonization events and gradual range expansion sometimes leading to longer and more complex routes around barriers [8, 28, 44, 65, 69, 78]. Speciation processes may further result from differential circannual timing of migratory programs and breeding segregation, where completely new migratory routes may be formed and kept separate in populations with limited gene flow [56, 65]. The question is how population-specific migratory routes evolve in birds with continuous breeding distributions that cover continental scales, such as the willow warbler *Phylloscopus trochilus* [27], for which suitable breeding as well as stopover habitats may be found across vast geographical areas. Although the accessible area is vast, the space-use patterns and migratory phenotypes are selected at the individual level, and hence, we should expect the encoded routes to be expressed by simple mechanisms in individual birds. Therefore, one may ask how the range expansion process to more distant breeding areas shapes the migratory routes of the individual migrants. First, is the gradually longer migratory routes guided by compass mechanisms that simply prolong the migratory path, or are new migratory routes formed as a sequence of different courses along the full migration (e.g. [38, 73])? Second, can the evolution of migratory routes used by individual birds be explained by one or several simple compass mechanisms [3], and if so, can we expect different compass mechanisms to be used at different latitudes [4]? We may further ask if adult birds follow the same route as they did as young inexperienced migrants and if they use additional cues for goal navigation during later migrations as may be expected (e.g. [74, 75, 77])? We do not, however, expect that experienced adult migrants will substantially alter the migration route in later migrations as compared to the first migration, but rather if possible to shorten the overall migration time between those sites [29].

In this study, we used miniature geolocators (GLS) (e.g. [68]) to track the extreme long-distance migration of willow warblers breeding in the most northeastern part of the breeding range in Russia to their presumed wintering areas in southern sub-Saharan Africa [27]. First, we were interested to determine the route choice by this population of willow warblers, the locations of stopover and wintering sites as well as the timing of migration. Second but foremost, we used these routes that traverse ~ 140° longitudes and ~ 80° latitudes to evaluate if the migration could be explained by a gradual increase in distance of a preferred route, based on a simple compass mechanism that relies on celestial or geomagnetic information [3, 4]. We therefore set up the following alternative hypotheses that willow warblers expanding to this easternmost part of the range would follow: 1) an autumn migratory route, explained by a single compass mechanism [3], or 2) a route, which involves directional shifts, with two (or more) distinct migratory courses differing from each other and expressed in sequence (e.g. [38, 73]). The first mechanism may allow for gradual range expansion that can include route reversal, in which the same compass mechanism guides birds along the route during autumn and spring. The second alternative may support a more complex evolutionary process in which migration to more distant breeding areas is incorporated in the program as a sequence of directional route shifts. Such shifts may be a result of range expansions from historically used breeding areas or stopover sites to the current breeding area, and may, or may not, be reversed in autumn to reach the wintering range. We expected the adult willow warblers to follow similar routes as they did during their first migration, but that they may have incorporated additional cues for navigation as compared to their first migration (e.g. [62, 74, 75, 77]). We further predicted that any course change or fueling episode at prolonged stopover might be triggered by geomagnetic information [16, 31].

## Results

### GLS tracking data

**Fig. 1:**
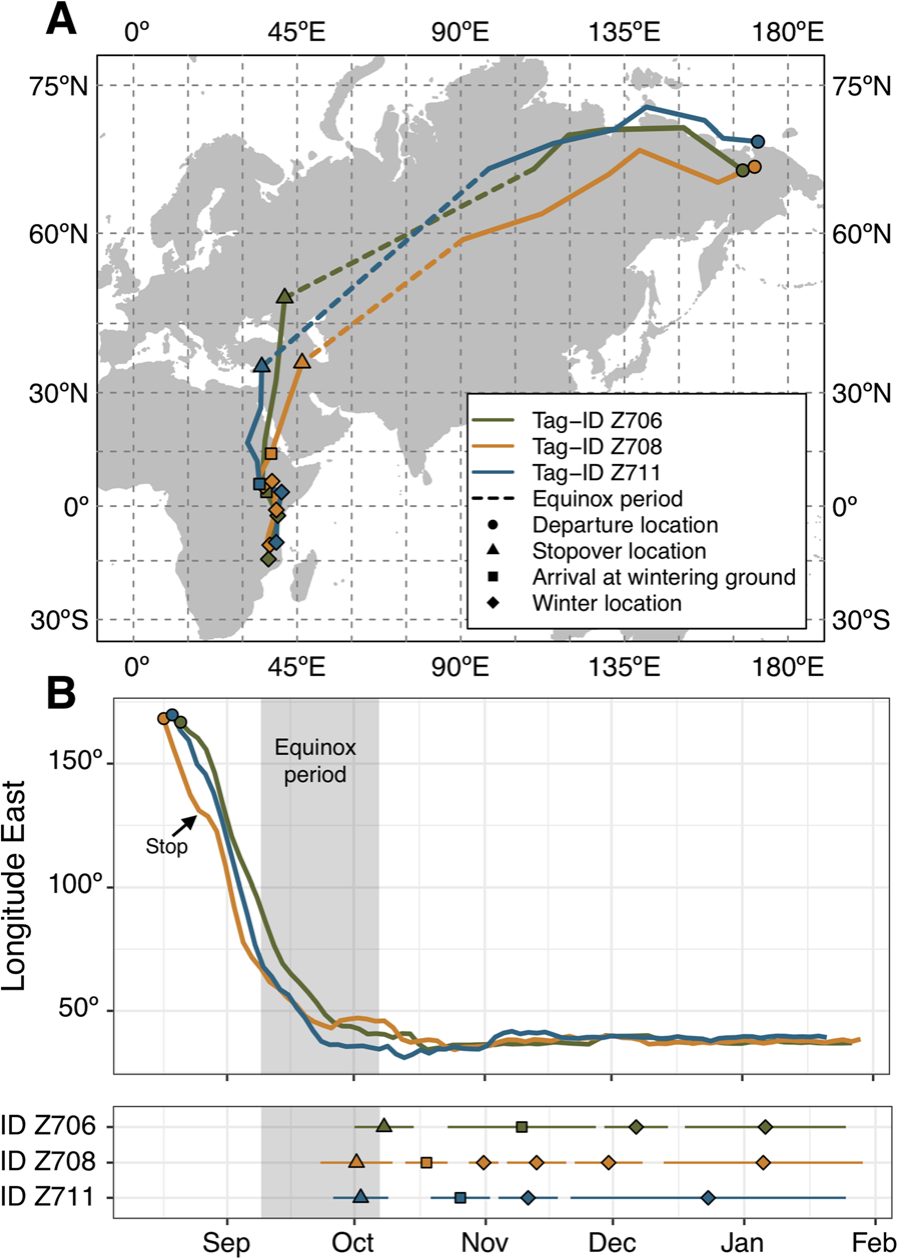
**a** Autumn migration routes and stopover sites of three willow warbler males (color coded) tracked by GLS loggers from northeast Russia to southeast Africa in 2016–2017. The autumnal equinox period (2 weeks before and after 23 September), is indicated as a broken line for each track. Map is in Mercator projection with 15° grid. **b** Autumn movements in relation to longitude as recorded by GLS for three male willow warblers migrating from Far East Russia to sub-Saharan East Africa. Indicated are departure from the breeding area (circles) and stopover sites and wintering areas (lower panel) including duration of the stops (horizontal lines). The autumnal equinox period is illustrated as shaded area. A short stop lasting 4 days for Z708 is marked with an arrow

Geolocator data recorded from three male willow warblers all showed similar use and timing of migratory routes. All three males departed from the breeding sites 17 to 21 August 2016 and arrived at an intermediate stopover site located in southwestern Asia (or possibly in southeast Europe) about 5 weeks later; however, the exact locations are uncertain as this period coincided with the autumnal equinox (Fig. 1a). The birds arrived at this stopover area 22 to 30 September, and stayed for 13–17 days. The birds again departed on a direction generally towards south to an initial wintering area in east Africa where they arrived 10–22 October for a stay lasting 10–35 days (Fig. 1a). Thereafter they all continued farther to the south in southeast Africa and made intermediate stops (1, 1 and 3; Fig. 1b) along the way to the last recorded wintering site in Tanzania and Mozambique, arriving 20 November, 12 and 17 December. After arrival at the last wintering area and a migratory route covering > 13,000 km from the breeding site, the batteries of the geolocators failed in the end of January, and we were unable to track any further movements. The GLS loggers registered light data for 190–199 days.

The willow warblers initially followed a northwest migratory route (one individual potentially first went south, Fig. 1a) when leaving the breeding area, and after crossing the initial longitudes (140–150°E), the routes were gradually directed more towards west and southwest until the birds reached the first stopover site (Fig. 1a). The end of this initial part of the migration occurred during the autumn equinox, when we lack latitude data for ca 4 weeks (22 September±14 days), and thus for this part of the migration we cannot identify the exact route. However, the crossings of longitudes, suggest a slow but continuous migration, with one bird showing a potential stop lasting only a few days (Fig. 1b). After the main stopover period in southwest Asia, the birds changed migratory route to south, along which they reached the wintering sites in Eastern Africa. The passage farther south in East Africa, followed at a very narrow longitudinal range for all birds (Fig. 1b).

Based on the GLS data we calculated the speed of migration along the initial part of the migratory route (excluding the fueling phase before start of migration) to be on average 178–286 km/day (initial 17–22 days of migration), as calculated for the great circle route distance [43]. In one of the birds, tracking data suggest a 3–4 day stop (65.88°N, 129.72°E), but for the other two birds we could not confirm any stop longer than 1 day prior to arrival at the stopover site in southwest Asia (Fig. 1a). The speed of migration (excluding fueling prior to departure, and thus overestimating the flight relative to stopover time) corresponds to birds spending 22–36% of their time per day in directional flight along the migratory route, assuming an air speed of 9.5 m/s [25] and movement in still air. The birds completed their migration from the breeding areas to the first sub-Saharan stopover site in 93–118 days.

### Route simulations

**Fig. 2:**
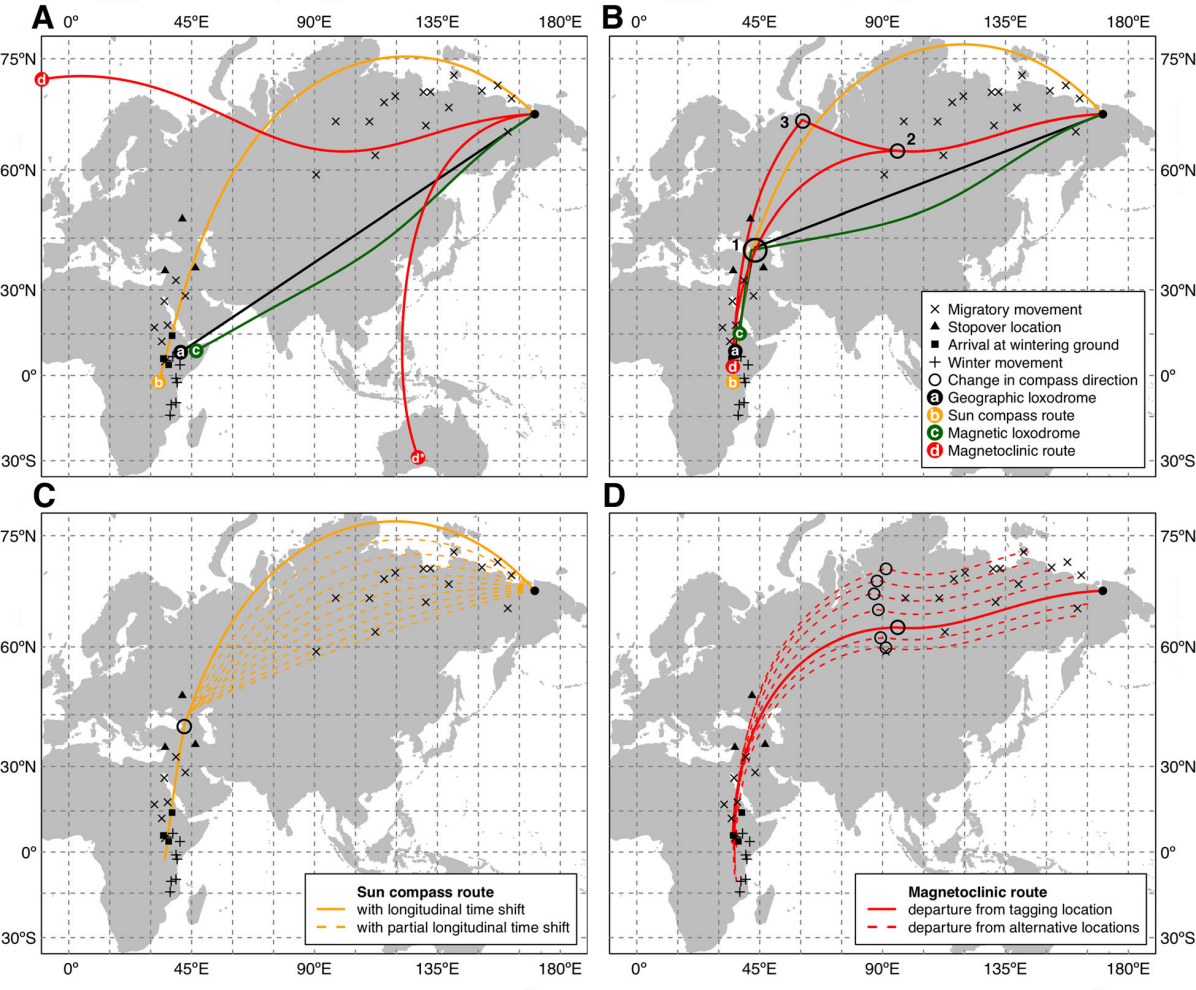
Simulated autumn migration routes of Willow warblers using alternative compass mechanisms and aiming to the wintering ground (first winter stop) through: (**a**) direct route or (**b**) an intermediate goal area (stopover region). In panel (A) only the sun compass route (b) crosses the identified stopover region (filled black triangles) and neither of the two possible solutions for the magnetoclinic route (d and d*) could reach the wintering area (see text). In panel (B) all routes have a solution that bring birds to the stopover region before switching the compass direction toward the wintering ground (black circle 1). However, for the magnetoclinic route the switch must occur earlier and at higher latitudes (between black circle 2 and 3) than for the other compass mechanisms (black circle 1). **c** A sun compass can produce alternative routes when the bird’s internal clock partially adapts to the local time (i.e. partial longitudinal time-shift), but an intermediate goal area would still be necessary to cross the stopover region. **d** Alternative magnetoclinic routes can be generated from locations with alternative geomagnetic inclination values (i.e. different starting points) for which a change in compass on route must always occur not earlier than 85—95 °E longitude. For all the panels, departure location (filled black circle) is the breeding area where birds where tagged. GLS data for the three birds tracked in this study are also reported. Maps are in Mercator projection with 15° grid and all simulated routes are 12,000 km except the northerly magnetoclinic route (d) in panel (A) that is 7750 km

In both direct route and intermediate goal routes, the geographic loxodrome (constant compass course relative to geographic north) and the magnetic loxodrome (constant compass course relative to magnetic north) did not coincide with the observed migratory routes, and both always lay much more south with respect to GLS positions obtained from the tracked willow warblers (Fig. 2a, and b). Thus, these routes could be ruled out and will not be discussed further. Only the simulated routes using either the sun compass or the magnetoclinic mechanisms were close to the observed autumn migratory routes in the three willow warblers.

The sun compass route was the only one crossing the stopover region by both a direct route (Fig. 2a), and by following an intermediate goal route (Fig. 2b). Furthermore, both simulated sun compass routes had northwesterly departure directions (315° and 319°, respectively) in line with the indication of our GLS data (see above). The sun compass route was, however, mainly north (up to thousands of km, see further below) of the GLS tracks including intersecting a stretch over the Arctic Ocean during the initial part of the migration.

The magnetoclinic route failed in the direct-route simulation (Fig. 2a) because there is no solution that can join departure and destination locations without a change in compass direction along the route. This is, however, expected by the vector-navigation mechanism proposed by Kiepenheuer [45] when applied to the autumn migration of birds breeding in the high arctic tundra in northeast Russia and wintering in Africa (see also [4]). Due to the extreme longitudinal displacement, any magnetoclinic route with an apparent angle even slightly higher than the local inclination will quickly turn toward south and, in our example, will end in Australia (Fig. 2a). On the other hand, if the bird keeps the apparent angle equal to the local inclination, the resulting route will be forced to follow the isocline and never descend below latitude 63° N (northerly magnetoclinic route in Fig. 2a). Hence, we will only discuss the magnetoclinic case when a change in compass direction occurs along the route, as proposed by Kiepenheuer [45]. In our case, we simulated that the birds follow an initial course by keeping the apparent inclination angle equal to the magnetic inclination present at the departure location (+ 77.9°), and after a certain distance travelled, they switch to an apparent inclination slightly larger than the initial + 77.9°. This change in the apparent inclination angle would allow the birds to leave the northerly latitudes and head toward the stopover locations and further south to the wintering ground (Fig. 2b). Since the change in direction can occur at multiple locations, we simulated the two most extreme scenarios: the earliest possible along the route (after 3500 km around 95° E) and the latest possible (after 5200 km around 60° E) that will bring the birds to the same destination (initial wintering site) in east-Africa after a total of 12,000 km (Fig. 1b). However, in autumn the willow warblers will continue for at least ca 1000 km longer to reach their second and third wintering site in southeast Africa.

## Discussion

Our GLS tracking data reveal a consistent timing and choice of autumn migratory route by the tracked male willow warblers breeding in their most northeastern part of the range in Russia [27]. The followed routes show predominant migration across land, and initial slow migratory speeds, when the birds appear to mix flight segments (22–36% of the time, excluding initial fueling time prior to departure) and stops corresponding to a ratio of flight and stopover time (fueling and resting) near 1:7 as predicted by Hedenström and Alerstam [35]. This is in agreement with observations that willow warblers carry minimal fat loads during the initial phase of autumn migration [49].

We investigated whether any alternative compass mechanism based on celestial information [11] or information from the geomagnetic field [45] could explain the observed autumn migratory routes in willow warblers breeding in the eastern part of the range. Both the geographic and magnetic loxodromes could be ruled out, as they both were much to the south of the observed tracks (Fig. 2a, and b). We found, notwithstanding the more acceptable results, that both the sun compass route and the magnetoclinic route gave results which disagreed with the recorded GLS positions while the birds were crossing the interior north of Russia (see longitudes 90°—175°E in Fig. 2b). Overall, the sun compass route was more to the north and would require flights partially crossing the Arctic Ocean during the beginning of the migration, which was clearly not followed by our GLS tracked birds. On the other hand, the magnetoclinic route was more to the south at the beginning of the migration (150°—175°E), and came closer to the recorded GLS locations later in the migration (90°—150°E, Fig. 2b). The distances from GLS locations were similar (range in longitudes 90°—175°E) for the sun compass (67—1522 km) and the magnetoclinic route (139—699 km), with no difference in the means (paired t-test, t (14) = 1.559, *p* = 0.141). However, we also found that both the sun compass and magnetoclinic route could lead to more realistic routes with some simple additional assumptions.

Indeed, the simulated sun compass route assumes that the bird’s internal clock is set to the departure location time and never adjusts with local time during the migratory flight leading to slightly curved routes as the longitudinal meridians are crossed [11]. However, it took about 5 weeks for our willow warbles after departure from the breeding sites to arrival at the first prolonged stopover in southwestern Asia. During this initial migratory phase with only 22–36% flight time per day and night, we expect the birds to land to rest and refuel along the route [35]. For a complete clock re-synchronization with local time, it has been shown for birds that a minimum of 3 to 5 days is needed [66], and therefore we could expect a slow drift of the internal clock toward the local time as the bird cross longitudes on the initial part of the migration. In this situation, the route taken by the birds would lie more southerly, and thus more in line with GLS locations observed in this study.

We produced alternative sun compass routes adding only a partially longitudinal time-shift to the mechanism proposed by Alerstam and Pettersson [11]. Those routes are presented in Fig. 2c, and show how moving from a 100% to a 10% longitudinal time-shift compensation of the sun compass mechanism, changes the shapes of the routes and displace them towards south closer to our GLS tracks. We simplified our simulation by adding a constant time-shift compensation along each route because little is known about how the internal clock in passerines adjusts to local conditions during long migrations [7]. In our willow warblers starting their migration from 20 August at 67°N, the ratio between daylight and darkness is 17:7 h, providing good opportunity to adjust the internal time sense along the way. In fact, we may expect a temporal shift to occur, since it is not yet known if birds are able to keep a clock synchronized at the starting point for sun compass orientation, and adjust their time sense at the same time [71]. Flights without time adjustments to local time is what would be required for the sun compass mechanism to guide birds along approximate orthodrome routes as proposed by Alerstam and Pettersson [11], and which was observed in high-arctic waders ([13, 14]; cf. [4]). The clear outcome of the simulated sun compass routes with partial longitudinal time-shift is that this mechanism requires an intermediate goal-area to be located in the observed stopover region combined with a successive change in compass direction before heading to the wintering grounds in east Africa (Fig. 2c). The use of a true sun compass mechanism, but with time resetting as outlined above, would have enabled willow warblers to expand their breeding range to the most eastern part of Siberia if they had evolved a migratory strategy to complete their migration with two different courses expressed in succession.

Similarly, for the magnetoclinic route a simple change in departure location could confer the route a more realistic shape that would better fit the GLS locations. In fact, as discussed above, the first leg of 3500—5200 km of the magnetoclinic route follows the isoclinic line of the departure location (Fig. 2b). Hence, if birds would move slightly to the north or south before departure, the entire first leg of the migration would shift to higher or lower latitudes, and thus being closer to our GLS data recordings. This is shown in our additional magnetoclinic route simulations with departures from different locations (Fig. 2d). Interestingly, all simulated magnetoclinic routes would require the earliest change in compass direction (i.e. change in apparent inclination angle as discussed above) around the 90°E longitude (Fig. 2d). This strategy would not require a different compass, but the birds could simply move to higher or lower values of magnetic inclination before starting the migration. Such strategy would make even more sense if considering that moving at higher latitudes the distance needed to cross meridians would be shorter and could thus provide a faster migratory journey. Whether our willow warblers indeed do so, is hard to evaluate due to the low level of precision in GLS tracking data at hand, but may be revealed in the future by alternative tracking technology. In particular, it would be of interest to have more detailed tracks of the first days of migration to evaluate if there are any pre-migratory movements before the start of the long migration.

Already in his initial presentation of the magnetoclinic route hypothesis, Kiepenheuer [45] noted that for forest living birds in the Palearctic-African migration system, this route would generally lead them across suitable habitats. The individuals we tracked started their migration by moving NNW, and thereafter they continued west across arctic tundra mainly north of the taiga forest belt. More recent work by Bolshakov [22, 23] support the suggestion that birds circumvent large barriers in the region showing that landbird migrants avoid passage of arid areas in Kazakhstan and central Asia, but concentrate in forested regions.

In this study we were able to track the migration of adult willow warblers, which has already performed at least one autumn migration. Although, we cannot rule out that adult birds use a different route than the juvenile birds, we expect them to most likely use the same route or a slightly more efficient route to reach the non-breeding destinations in a shorter time as migration speed is often higher in adult birds as compared to juvenile birds [29]. Adult birds may use additional cues for goal navigation [62, 74, 75, 77] either to correct for displacements along the route [9, 64] or to pin-point known stop-over sites or both. Whether our adult willow warblers use a moving goal-area strategy [64] along the migration route including course corrections or follow a compass route and only navigate, i.e. correct for any displacements near the resident areas is hard to say. It cannot be ruled out that the warblers were able to define their position and correct for potential displacements along the route as the migration speed was generally low in the first part of the migration. Our data suggest a migration strategy with intermediate flights and stationary periods mixed on a daily basis during which time reorientation may be achieved. However, if we expect adult birds to include map information in their migration program by individual experience during their first migration [74, 75, 77], we would expect them to keep to those learned route-specific “signpost cues” in following migrations. Magnetic gradients have been suggested to form geomagnetic bi-coordinate maps extending over substantial range [52, 70], which would likely be functional over the migration distances and most of the range our willow warblers from east Russia cover in autumn [24]. Thus, although our route simulations suggest that modifications of two compass mechanisms based on the sun and the geomagnetic field would be able to generate realistic migration routes as tracked for our adult willow warblers, we cannot exclude that these birds also used some sort of map navigation to keep those routes, possibly based on geomagnetic information [24, 70].

Many ring recoveries have shown that the northern European subspecies of willow warblers (*P. t. acredula*) share the same east African wintering ground as we have found for *P. t. yakutensis* [36]. Moreover, the two subspecies are genetically inseparable (F_st_ = 0) based on > 4000 SNPs located in the nuclear genome and in complete sequences of the mitochondrial genome [54]. Based on these observations it is reasonable to assume that they comprised one common refuge population during the last glaciation. Our study area might have been colonized by willow warblers during the Holocene climatic optimum between 6000 to 8000 years BP [53]. One can speculate that this refugium was located in southwest Asia (or southeast Europe), where our tracked birds made the first longer stop followed by a change in direction of migration. A gradual range expansion towards north and east from this refugium, across the 6000 km of its Eurasian breeding range, would however not require major genetic changes in their migratory program as the orientation mechanism here proposed would take the birds to the intermediate goal in southwest Asia, and we may expect a similar mechanism to work from other starting longitudes [45]. A magnetoclinic mechanism has been demonstrated to provide realistic routes during spring migration (for route simulations see, [4]) for Northern wheatears *Oenanthe oenanthe* breeding in Alaska and wintering in eastern Africa [15]. Data further support the possibility that willow warblers gradually expanded from this southwestern Asian refugium population, by following a sun-compass route in spring adjusting for the time-shift along the way. Tracking data from spring migration, however, will be needed to disentangle the two alternatives for our eastern willow warblers.

## Conclusions

The most likely compasses used by willow warblers during the autumn migration from northeast Siberia to southeast Africa are either based on the sun compass mechanism [11] or the magnetic compass based on the inclination of the magnetic field [45], but with some modifications. The same mechanism will function also in a reversed situation during spring migration, which has been investigated for Northern wheatears *Oenanthe oenanthe* breeding in Alaska and migrating to east Africa ([4], tracking data from [15]), providing support for use of a magnetoclinic route and the inclination compass Kiepenheuer [45]. For the sun compass to work, we assume the internal clock of the bird would partially drift toward the local time as the bird migrate west or east (slowly crossing longitudinal meridians), while for the magnetoclinic route we assume the birds start the migration from a different local geomagnetic inclination value than the breeding location. Furthermore, both mechanisms needed a change in the compass direction (or an intermediate goal area) to follow the real migratory tracks; for the sun compass a possible intermediate goal co-occurs with the main stopover region recorded, and for the magnetoclinic route between 60°E and 95°E longitude. At this point it is, however, difficult to disentangle which of the two mechanisms is used during the migration or if a hierarchical criterion exists between the two compasses [2, 57, 58], where one compass could be recalibrated by the other ([57], cf. [6]). Furthermore, we cannot rule out a situation where the birds may combine both mechanisms in their migration strategy. Still, we find it promising that a modified compass mechanism based either on the sun or the geomagnetic field, or a combination of the two, may explain the autumn migration route of the longest songbird migration. We conclude that both compass mechanisms will also function in a reversed situation in spring, but whether birds indeed reverse their routes in spring needs to be investigated. We furthermore suggest that future studies should collect tracking data from birds inhabiting different parts of the high arctic breeding range, to investigate if any of the alternative mechanisms highlighted here may explain migration from different locations, or tracking data will support a combined strategy.

## Methods

### Study species

The willow warbler (*Phylloscopus trochilus*) is a songbird migrant inhabiting extensive parts of northern Europe and Asia for breeding and wintering in sub-Saharan Africa [27]. Willow warblers use a variety of forested habitats for breeding and may occur in high numbers in parts of their breeding range, being the most numerous breeding bird in e.g. Sweden [61]. Three subspecies occur across its range *P.t. trochilus* and *P. t. acredula* meeting in a hybrid zone in central Sweden [19, 20, 26], and *P.t. yakutensis* occurring farther to the east in Russia [27, 46]. *P.t. trochilus* and *acredula* are known to have different migration phenotypes, route choices and winter destinations in west and southeast Africa, respectively [19, 20, 26, 47], while much less is known about the route choice and winter destinations of *P.t. yakutensis*.

### Study site and field work

Field work was conducted on Ayopechan island in the Chaun delta in northeast Russia (68.81° N, 170.62° E) during June and July in 2016 and 2017. Habitat at the study site was typical arctic tundra with low vegetation and many thermo-karst lakes [67]. Willow warblers of the eastern subspecies (*Phylloscopus trochilus yakutensis*) breed in thick *Duschekia* and *Salix* shrubs growing along river shores. Territorial, breeding males were attracted with song playback and trapped with mist nets. Geolocators (Migrate Technology LTD, Intigeo-W30Z11-DIP 12x5x4mm, 0.32 g) were fitted on 29 males with leg loop harnesses made from a 1 mm nylon string [59]. The mass of the geolocator in no case exceeded 5% of the body mass of the bird. The geolocators had a maximum expected battery duration of 8 months and were programmed to start 15 July. In summer of 2017 the breeding sites were revisited and three geolocators were successfully retrieved (Table 1). One of the returning birds had lost the geolocator.

**Table 1:** Number of ringed and GLS tagged male and female willow warblers in 2016 and the number of recovered birds in 2017

	Ringed 2016	GLS 2016	Recovered 2017 (ringed)	Recovered 2017 (GLS)	Total recovered
Males	6	29	1	4	5 (14.3%)
Females	10	–	2	–	2 (20%)

### Preparation of light logger data

All light loggers had terminated recording between 20 and 29 January and data were extracted by Migrate Technology LTD. Positions were estimated using the GeoLight package [50] in R (R Core Team 2014). We used a threshold of 3 lx to estimate sunset and sunrise transitions. We removed outliers using the loessFilter function in GeoLight with a k-value of 1. To estimate the sun elevation angles corresponding to the selected light threshold, we calibrated the data using a Hill-Ekström calibration [51]. This method takes advantage of the fact that the correct sun angle leads to the most stable latitudes during a stationary period, especially evident near the equinoxes. We therefore performed this calibration at the first longer stopover just after the autumn equinox (squares in Fig. 1a). The resulting sun angles were − 0.7, − 1.2 and − 0.5. We removed latitudes for a minimum of 12 days on either side of the autumn equinox due to the uncertainty of latitude estimation during this period.

Stationary periods were found by manually identifying segments of stable longitude and latitude (when available). Only stationary periods separated by more than 2° longitude or 5° latitude that lasted for a minimum of 5 days were considered due to the uncertainty in this type of geolocation; however, we checked the data for indications of shorter stopovers during the long initial migration leg. Stopovers located nearby were aggregated, and shorter stopovers were considered part of the movement phase. Departure date was considered the last day within 1° degree of the stopover average of both longitude and latitude and arrival the first day within 1° of the average. For illustration purposes, we plotted 3-day location means during the movement phases in Fig. 1 and in relation to longitude in Fig. 2. Movement phenology for each bird is given in Additional file 1: Table S1.

Speed of migration (km/day) was calculated based on the GLS data, from the latitude and longitude positions and time passed between those locations, as calculated for a great circle route distance [43].

### Route simulations

We simulated the autumn migration of the willow warblers from the breeding area in eastern Siberia to wintering grounds in central-east Africa assuming four vector-navigation mechanisms following the approach in Åkesson and Bianco [3]. Each mechanism is based on a proposed compass used by birds for navigation that generates a predictable route as follows: (a) geographic loxodrome route, generated by a star compass [30]; (b) sun compass route, using the bird’s internal clock to predict the apparent motion of the sun and taking into account the longitudinal time-shift according to Alerstam and Pettersson [11]; (c) magnetic loxodrome route, with constant bearing relative to magnetic North; and (d) magnetoclinic route, keeping constant the apparent angle of magnetic inclination according to Kiepenheuer [45]. For more detailed definitions, see Åkesson and Bianco [4]. We used as departure location the breeding area where birds were tagged (68.78 °N, 170.65 °W), and routes of length 12,000 km leading to the initial sub-Saharan wintering site (Fig. 2). For each compass, we selected the departure direction of the route that satisfied either of two conditions: final location was the closest possible to destination in Africa (Fig. 2a), or an intermediate goal located in the central position of the stopover region identified by the geolocators (Fig. 2b). In the latter simulations, all routes required a change in compass direction (Fig. 2b).

## Additional file

Additional file 1: **Table S1.** Migration phenology for the three male willow warblers (Z706, Z708, Z711) tracked by GLS from eastern Russia to southeast sub-Saharan Africa. Dates are given as Julian dates and latitude and longitude (±SD) are given as degrees. (DOCX 17 kb)
