# Supplementary material for: Ten grams and 13,000 km on the wing – route choice in willow warblers Phylloscopus trochilus yakutensis migrating from Far East Russia to East Africa
Source: Mov Ecol. 2018 Oct 15;6:20. doi: 10.1186/s40462-018-0138-0 (PMC6191995; doi:10.1186/s40462-018-0138-0)
Supplement: Supplementary file 1 — Table S1. Migration phenology for the three male willow warblers (Z706, Z708, Z711) tracked by GLS from eastern Russia to southeast sub-Saharan Africa. Dates are given as Julian dates and latitude and longitude (±SD) are given as degrees. (DOCX 17 kb) [file 40462_2018_138_MOESM1_ESM.docx]

**Supporting data**

Additional file 1: Table S1 Migration phenology for the three male willow warblers (Z706, Z708, Z711) tracked by GLS from eastern Russia to southeast sub-Saharan Africa. Dates are given as Julian dates and latitude and longitude (±SD) are given as degrees

| Z706 | Arr | Dep | Days | lon | lat | lonsd | latsd |
| --- | --- | --- | --- | --- | --- | --- | --- |
| Breeding | 220 | 234 | 17 | 167,5608 | 67,48751 | 1,643902 | 0,892432 |
| Stop1 | 274 | 288 | 15 | 41,58813 | 49,60218 | 1,17775 | 4,823082 |
| Winter1 | 296 | 331 | 36 | 36,58466 | 3,916379 | 0,549127 | 1,474066 |
| Winter1_1 | 333 | 348 | 16 | 39,69592 | -2,6139 | 0,822638 | 4,044459 |
| Winter2 | 352 | 25 | 39 | 37,12294 | -14,4571 | 0,481416 | 1,907386 |
|  |  |  |  |  |  |  |  |
| Z708 | Arr | Dep | Days | lon | lat | lonsd | latsd |
| Breeding | 210 | 230 | 21 | 170,8292 | 67,86536 | 1,94363 | 0,512265 |
| Stop1 | 266 | 283 | 18 | 46,37833 | 36,57885 | 0,803628 | 6,307026 |
| Winter1 | 286 | 296 | 11 | 37,84242 | 14,31892 | 0,795686 | 3,637749 |
| Winter1_1 | 301 | 308 | 8 | 35,69641 | 5,515489 | 0,558683 | 3,831042 |
| Winter1_2 | 310 | 324 | 15 | 38,10498 | 6,828926 | 0,838832 | 3,709565 |
| Winter1_3 | 326 | 342 | 17 | 39,2881 | -1,05515 | 0,882115 | 3,498582 |
| Winter2 | 347 | 29 | 48 | 37,4984 | -10,67 | 0,773846 | 2,828433 |
|  |  |  |  |  |  |  |  |
| Z711 | Arr | Dep | Days | lon | lat | lonsd | latsd |
| Breeding | 210 | 232 | 23 | 171,7379 | 70,33299 | 1,674736 | 0,596054 |
| Stop1 | 269 | 282 | 14 | 35,28108 | 35,72983 | 0,84683 | 6,07864 |
| Winter1 | 292 | 306 | 15 | 34,64948 | 6,077333 | 1,105082 | 3,874195 |
| Winter1_1 | 308 | 322 | 15 | 40,74034 | 3,851688 | 0,874401 | 2,666519 |
| Winter2 | 325 | 25 | 66 | 39,26029 | -9,95592 | 0,620592 | 2,339836 |
